# Case Report: A Case of Congenital Nephrogenic Diabetes Insipidus Caused by Thr273Met Mutation in Arginine Vasopressin Receptor 2

**DOI:** 10.3389/fped.2021.707452

**Published:** 2021-07-15

**Authors:** Li Huang, Lina Ma, Linjing Li, Jiajia Luo, Tianhong Sun

**Affiliations:** ^1^Department of Pediatric Nephrology, Lanzhou University Second Hospital, Lanzhou, China; ^2^Department of Nephrology, Gansu Children's Hospital, Lanzhou, China

**Keywords:** congenital nephrogenic diabetes insipidus, type 2 arginine vasopressin receptor 2, missense mutation, X-linked recessive inheritance, water reabsorption

## Abstract

Congenital nephrogenic diabetes insipidus (CNDI) is a rare hereditary tubular dysfunction caused mainly by X-linked recessive inheritance of *AVPR2* gene mutations. Pathogenic genes are a result of mutations in *AVPR2* on chromosome Xq28 and in *AQP2* on chromosome 12q13. The clinical manifestations of CNDI include polyuria, compensatory polydipsia, thirst, irritability, constipation, developmental delay, mental retardation, persistent decrease in the specific gravity of urine, dehydration, and electrolyte disorders (hypernatremia and hyperchloremia). Herein, we report a rare case of CNDI caused by an *AVPR2* mutation in a 2-year-old Chinese boy who had sustained polyuria, polydipsia, and irritability for more than 20 months. Laboratory examinations showed no obvious abnormality in blood sodium and chloride levels but decreased urine osmolality and specific gravity. Imaging findings were also normal. However, genetic analysis revealed a C > T transition leading to T273M missense mutations in AVPR2. We provided the boy a low-sodium diet and administered oral hydrochlorothiazide and indomethacin for 1 month, after which his clinical symptoms significantly improved. This case report suggests that CNDI is characterized by pathogenic T273M missense mutations alone and expands our understanding of the pathogenesis of CNDI.

## Background

Congenital nephrogenic diabetes insipidus (CNDI) is a rare hereditary tubular dysfunction characterized by impaired renal tubule reabsorption of water. In this condition, renal distal convoluted tubules and collecting tubules are not sensitive to arginine vasopressin (AVP); therefore, the kidney cannot concentrate urine and discharges a large amount of unconcentrated urine (1, 2). The clinical signs of CNDI include polyuria, compensatory polydipsia, thirst, irritability, constipation, growth retardation, mental retardation, persistently decreased urine specific gravity, dehydration, and electrolyte disorders (hypernatremia and hyperchloremia) (3, 4). Most CNDI cases (about 90%) are caused by arginine vasopressin receptor 2 (AVPR2) gene mutations, leading to X-linked recessive hereditary diseases. Approximately 10% of CNDI cases are caused by aquaporin 2 (AQP2) gene mutations (9%, autosomal recessive inheritance; 1%, autosomal dominant inheritance) (2). CNDI patients develop urine concentration deficits in the first week after birth, but in a cohort study, the average age of CNDI onset was around 3 months (5). It is difficult to detect polyuria and polydipsia in infancy. The most common symptoms are fever, irritability, feeding difficulties, constipation, weight loss, and slow development, which may lead to growth retardation, mental retardation, etc. (6). Because of the rarity of this condition and because the symptoms are not typical in young children, the chances of missed diagnosis or misdiagnosis are high.

In this report, we discuss a case of CNDI caused by an *AVPR2* missense mutation in a 2-year-old Chinese boy. The chief complaints were polyuria, polydipsia, irritability, and stunting. Laboratory and imaging examinations showed no obvious abnormality in serum sodium and chloride levels, and the urine osmolality and specific gravity were decreased. Genetic analysis revealed a T273M missense mutation in *AVPR2*, confirming the diagnosis of CNDI in the patient.

## Case Presentation

A 2-year-old boy was admitted to the hospital with intermittent low-grade fever, polydipsia, polyuria, anorexia, no abnormal crying, unconscious smile, and irritability that had been present for more than 20 months. The patient did not have any siblings and was born at 40 weeks of gestation through vaginal delivery without significant prenatal complications. He had been breastfed and provided supplementary feeding on demand. He had also received the appropriate vaccinations on time. No significant growth retardation was noted before admission. The patient had xerostomia, polyuria, and polydipsia with >4,000 mL liquid intake daily. The patient's parents have married consanguineously, the mother has no previous history of abortion, stillbirth, etc. The grandfather of the parents' family had polyuria.

Physical examination at admission showed blood pressure of 83/46 mmHg, respiratory rate of 20/min, heart rate of 100/min, and body temperature of 36.1°C. The boy's bodyweight and height were 12 kg and 90 cm, respectively, both values within the normal range of the same age group(P25–P50 and P50–P75, respectively). No obvious abnormalities in the heart and lungs were noted, and physiological reflexes were normal. However, Gesell Developmental Schedules confirmed developmental delay (7).

Laboratory examinations showed normal urine ion content. Urine specific gravity and urine osmolality were decreased, but blood osmolality and sodium and chloride ion levels in the blood were not abnormal (Table 1).

**Table 1:** Results of laboratory examinations and clinical manifestations before and after treatment.

	**Before treatment**	**After 1 month treatment**	**Reference range**
**Laboratory examinations**			
Specific gravity of urine	1.001	1.002	1.001–1.025
Blood osmolality (mOsm/kg)	285	286	280–310
Urine osmolality mOsm/kg H_2_O)	85	98	550–1,100
Blood sodium (mmol/L)	136	137	135–147
Blood chloride (mmol/L)	98	95	95–110
**Clinical manifestations**			
Daily volume of liquid intake (mL)	>4,000	~2,000	§
Daily volume of urine output (mL)	>3,000	~2,000	§
Frequency of urination (nighttime)	6–7	〈〈1	§
Body height (cm)	90	90	§
Body weight (kg)	12	12	§

Accessory examination showed normal sonography of the heart, liver, gallbladder, pancreas, spleen, and kidneys. A cranial MRI scan showed normal brain parenchyma. Further, to confirm our diagnosis, a water deprivation test was proposed. It is important to distinguish central diabetes insipidus (CDI) from nephrogenic diabetes insipidus (NDI) if the urine volume does not change much after the water deprivation test. Body weight, blood pressure, plasma osmotic pressure, electrolyte level, urine specific gravity, and urine osmotic pressure are analyzed. If the urine osmotic pressure reduces to <30 mOsm/kg H_2_O, the patient is administered desmopressin (1-deamino-8-D-arginine vasopressin) via nasal injection (10–25 μg) or subcutaneous injection (1–2 μg) for about 2–3 times, and the urine specific gravity, urine osmotic pressure, plasma osmotic pressure, and electrolyte level are measured at 30, 60, and 120 min after administration. In our patient, we observed a significant decrease in CDI symptoms but poor improvement in CNDI symptoms. Moreover, because of the patient's young age and intolerance to excessive thirst, the water deprivation test could not be conducted. In addition, central diabetes insipidus is caused by hypothalamus and neuropituitary developmental defects, and the MRI findings of the head of the patient revealed no abnormalities. Therefore, we had greater suspicions of nephrogenic diabetes insipidus, which is why we conducted a genetic examination.

**Figure 1:**
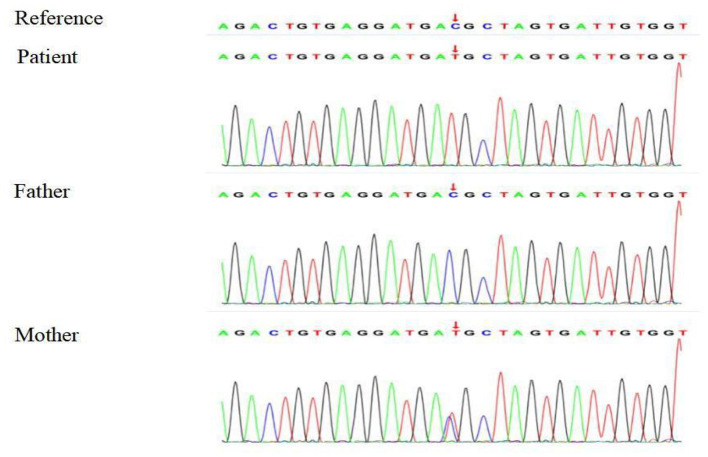
Sequencing analysis shows a heterozygous 818 C > T transition in exon 2 of *AVPR2* in the CNDI patient (Arrow, *middle*). A heterozygous 818 C > T transition was noted at the same position of *AVPR2* in the patient's mother (Arrow, *lower*).

Based on the clinical, laboratory, and imaging findings, the patient was initially diagnosed with NDI. Exome group tests were conducted for the patient and his parents to determine the genetic causes of potential NDI. Targets were enriched by using the xGen Research Exome Panel v2.0 whole exon capture chips (Integrated DNA Technologies, Inc., IA, USA) and sequencing on the Illumina NovaSeq 6000 System (PE150). Suspected mutations were confirmed by Sanger sequencing. The genetic test showed that the patient had a heterozygous C > T transition at nucleotide 818 (c.818C>T) in exon 2 of the *AVPR2* gene, leading to a missense mutation at amino acid 273 (p.T273M) of AVPR2. Notably, the patient's mother harbored a heterozygous mutation at the same location of *AVPR2*, whereas his father showed no mutation at the same location of *AVPR2* (Figure 1). Due to the consanguineous marriage of the parents and the lack of NDI symptoms in the patient's mother, we concluded that the mother was likely a carrier of the c.818C>T mutation, which was reinforced by the fact that the boy's grandfather had polyuria and polydipsia. Taken together with the clinical manifestations, the boy was diagnosed with CNDI caused by a missense mutation in *AVPR2*.

## Treatment

For preventing the development of a hyperosmotic state caused by hypernatremia and hyperchloremia, we mainly provided the boy a high-liquid sodium-limited diet, Furthermore, because the urinary output was low, hydrochlorothiazide tablets (5 mg, t.i.d.), and indomethacin enteric-coated tablets (1/3, t.i.d.) were administered. Potassium chloride granules (0.5 g, q.d.) were administered to prevent hypokalemia. After 1 month of treatment, the patient's clinical symptoms of thirst and decreased urinary output were markedly relieved. Electrolyte levels in the urine and serum were monitored monthly and followed up regularly.

## Discussion

Under physiological conditions, water balance is essential to maintain systemic homeostasis. The renal water balance is mainly regulated by the structure and transport function of the medullary loop in the kidney and by antidiuretic hormone and AVP secreted by the pituitary gland. The release of AVP is regulated and affected by multiple factors, among which humoral osmotic pressure (increased plasma osmotic pressure due to hypernatremia) and circulation volume (decreased blood volume i.e., hypovolemia) are the main inducers of AVP release. This physiological condition is called antidiurea. There are two AVP receptors, AVPR1 and AVPR2. AVPR1 receptors are distributed in vascular smooth muscles, whereas AVPR2 is mainly present in distal convoluted tubules and collecting ducts of the kidney, which belong to G protein-coupled receptors.

In the present study, we identified the X-linked inherited missense mutation c.818C> T (p.T273M), which has not been reported previously in China. CNDI is a rare disease with a prevalence of about 1:25000 (8). It can manifest at any age, the prevalence rate is unknown, and the incidence is higher in male infants than in female infants. Ouweland et al. first reported an *AVPR2* mutation in a patient with renal diabetes insipidus and a family history of X-linked recessive genetic mutation in 1992 (9). Joshi et al. studied 19 CNDI families that had *AVPR2* mutations in 2018. Analysis of the human gene database revealed that there are about 281 cases of X-linked renal insipidus caused by *AVPR2* mutations (10). X-linked CNDI mostly affects male patients, and heterozygous women can show different degrees of clinical symptoms (polydipsia, polyuria, etc.) and other severity is different, mainly caused by X- chromosome bias inactivation (11). According to foreign research statistics, roughly one in 250,000 men has an X-linked CNDI, and an estimated 400 individuals suffer from CNDI in Japan (12). A report from Canada showed that the prevalence of X-linked CNDI in Quebec, Canada, is about 8.8/100 million live births in men, which is six times higher than that in Nova Scotia and New Brunswick (13).

CNDI is a rare renal tubular hereditary disease caused by *AVPR2* or *AQP2* genetic mutation. The pathogenesis is a result of mutations in *AVPR2* on chromosome Xq28 and in *AQP2* on chromosome 12q13 (3). Most CNDI cases (90%) are reported in males, showing an X-linked recessive inheritance pattern, mostly familial, leading to the deficiency of AVPR2 in renal epithelial cells. The clinical symptoms vary greatly in the severity of polydipsia and polyuria (14). AVPR2 belongs to a class of G protein-coupled receptors (GPCRs), which are composed of 371 amino acids. It is a typical 7-transmembrane helix GPCR dispersed in renal tubules and regulates water metabolism *in vivo*. GPCRs are the most abundant type of cell membrane receptors, and their mutations can cause many diseases. AVPR2 mainly couples with Gs proteins to regulate the level of 3′-5′-cyclic adenosine monophosphate (cAMP). In nephrocytes, cAMP acts as a second messenger to activate protein kinase A (PKA), which phosphorylates AQP2 tetramer. The resultant exocytosis of AQP2 on the apical membrane of the cells of the renal collecting duct results in the reabsorption and concentration of urine. *AVPR2* has been determined to be located on the telomere band Xq28 in the long arm of the X chromosome; it includes three exons and two introns. In a previous study, CNDI patients did not respond to treatment with desmopressin, suggesting that cAMP signaling pathways are defective in CNDI patients (15).

*AVPR2* missense mutations (A89P, G107R, Q174R, V277A) affect the location of the corresponding receptors during mammalian evolution. Mutations in AVPR2 folding, transport, and functional residues are responsible for CNDI pathogenesis (16). At present, more than 600 diseases caused by mutations in GPCR family genes have been identified, including CNDI (17). Most variants have been reported in the transmembrane region. To date, 287 potential pathogenic mutations of *AVPR2* have been described in the human gene database (The Human Gene Mutation Database at the Institute of Medical Genetics in Cardiff; http://www.hgmd.cf.ac.uk), of which 177 (62%) mutations are missense mutations (18). In addition, about 25% of the mutations are frameshift mutations caused by nucleotide sequence deletion or insertion, 10% are non-sense mutations, 9% are large fragments; complex rearrangements, splicing mutations, or insertion mutations are rare (7, 19). About 70 *AQP2* mutations have been described, including 54 missense mutations, 4 splicing mutations, 9 minor deletions, 1 major mutation, and 2 minor insertions (18). Overall, *AVPR2* or *AQP2* mutations are the genetic basis for CNDI.

According to the function and subcellular localization of mutant proteins, *AVPR2* mutations are classified into five types. Type I mutations that interfere with correct receptor transcription, mRNA processing or translation, and accelerated protein degradation. Type II mutations, the most common type of mutation, are often caused by *AVPR2* mutations (missense, insertion, or deletion), resulting in *AVPR2* mutants with intracellular transformation defects, most of which remain in the endoplasmic reticulum (ER). Type III mutations mainly occur on the lateral basement membrane. They affect the expression of receptors on the plasma membrane and their ability to bind to or activate G proteins under AVP stimulation (IIIa subtype). When coupling with Gs protein is affected, AQP2 cell exocytosis is promoted. Type IV mutations have a low affinity for vasopressin. Class V mutants are mismatched into different subcells and mislocated within cells (20–22).

CNDI patients are born with defects in urine concentration and are prone to develop repeated low-grade fever, irritability, nausea, vomiting, feeding difficulties, slow weight gain, constipation, and other non-specific clinical manifestations (20). Therefore, CNDI is easily misdiagnosed. As the age increases, CNDI affects night sleep due to polydipsia, polyuria, and thirst, which seriously affect the quality of life of children. The inability of CNDI patients to concentrate urine and the lack of self-compensatory regulation of water supply or temporary loss of water can lead to dehydration, dry skin, skin inelasticity, eye socket depression, and anterior fontanelization depression, which can lead to electrolyte disorders such as hypernatremia, hyperchloremia, and the risk of severe dehydration.

Therefore, diabetes insipidus is considered if the patient shows unexplained fever, wet weight of diapers, feeding difficulties, constipation, low weight gain, and slow development in the post-natal and infancy stages; polyuria, polydipsia, and irritability in children and adults; and exclusive infections without other significant abnormalities and antibiotic treatment failures. CNDI results from *AVPR2* or *AQP2* mutations. Since there is no difference in the clinical symptoms of CNDI caused by *AVPR2* and *AQP2* mutations, it is difficult to establish a clear family history; therefore, DNA sequencing is strongly recommended to obtain the correct genetic diagnosis (23). Since *AVPR2* and *AQP2* are relatively small genes, their sequences can be easily detected, so that amniotic cells, chorionic villi samples, or umbilical cord blood samples from fetuses in the perinatal or prenatal period can be tested if the family members are suspected to be carriers of *AVPR2* and *AQP2* mutations (19). For patients with a suspected diagnosis of CNDI, it is recommended to determine their genetic characteristics as soon as possible to develop relevant treatment strategies.

Current treatment includes administration of hydrochlorothiazide (2–4 mg/kg/day) combined with amiloride (0.3 mg/kg/day), which reduces urine volume by 50%, is tolerated well, and can reduce the occurrence of hypokalemia (24, 25). Amiloride is prone to cause persistent nausea, and in such cases, it can be replaced with indomethacin. Different clinicians may suggest different treatments and diet plans for CNDI patients. Most clinicians (93%) prescribe thiazide for treating CNDI, whereas 62, 55, and 43% prescribe amiloride, non-steroidal anti-inflammatory drugs, and indomethacin, respectively, according to a survey (26). The most commonly used treatment regimen is hydrochlorothiazide (2–4 mg/kg/day) combined with indomethacin (0.75–1.2 mg/kg/day), which can effectively reduce urine volume and further reduce water excretion (27, 28). In this study, hydrochlorothiazide combined with indomethacin was used. After 1 month of treatment, the symptoms of thirst and polyuria and the number of urination episodes at night significantly reduced. Although this treatment can relieve clinical symptoms of congenital CNDI, it cannot address the inability of the patient to produce concentrated urine. Long-term use may cause electrolyte disturbance, gastrointestinal dysfunction, and renal function damage, which may affect the quality of life of children. Hence, it is now crucial to find more effective treatments for CNDI patients.

Thus, far, clinical strategies for treating CNDI include limiting sodium intake, ensuring sufficient fluid intake, minimizing water excretion, and correcting hypernatremia- and hyperchloremia-induced hyperosmotic status. Many recent studies have explored novel treatment strategies, and the proposed treatment usually involves the following aspects: direct activation of cAMP signaling pathways in primary renal cells, AQP2 activation via bypassing of AVPR2 signal transduction pathways, restoration of the impaired receptor function, and direct improvement of AQP2 function (20). Restoration of cAMP signaling pathways disrupted by *AVPR2* mutations using chemical chaperones, glycerol, and dimethyl sulfoxide, facilitates the correct folding of mutant proteins (unknown mechanism), enabling the misfolded proteins to escape from the ER and be expressed normally (29). Non-peptide AVPR2 antagonists, such as SR49059V1a receptor non-peptide antagonist, can be combined with type II AVPR2 mutants in the ER to maintain their stable conformation, reduce protein misfolding, and normalize their expression on the cell membrane and surface (30). Non-peptide AVPR2 agonists can enter cells and bind and activate AVPR2 mutants, but they cannot stabilize their conformation. They act via the G protein-cAMP complex pathway to activate intracellular AVPR2 mutants, which results in cAMP activation and the phosphorylation and expression of AQP2 in the apical cell membrane, thereby reducing CNDI pathogenesis (31). β3-Adrenergic receptors also promote AVPR2 expression in most renal units, including the ascending branch of the loop, the cortex, and the extramedullary collecting duct (32). The calcium signaling pathway is also considered to be a therapeutic target for CNDI, and calcineurin is a key molecule in AQP2 activation. Calcineurin can directly activate arachidonic acid to produce vasopressin, and calmodulin and calcineurin can effectively induce AQP2 activation without vasopressin. The Wnt5a -calcium-calmodulin-calcineurin signaling is reported to induce AQP2 phosphorylation, transport, and mRNA expression (12, 33). cAMP-induced PKA activation is considered to be the main mechanism of AQP2 phosphorylation and transport in the vasopressin signaling pathway (34). Selective prostaglandin receptor agonists, metformin, epidermal growth factor receptor inhibitors (e.g., erlotinib), phosphodiesterase inhibitors (e.g., sildenafil), statins, calcitonin, and pancreatic hormone receptor agonists have been reported to be useful in treating CNDI. However, the clinical safety and efficacy of these new drugs need to be evaluated in further clinical trials.

This study presents a case of X-linked recessive hereditary CNDI that was caused by a C >T transition mutation of *AVPR2* leading to a missense T273M variant. It suggests that threonine 273 is essential for *AVPR2* function. The Discovery of a new mutant gene adds new information to the human gene mutation database. According to research on genotyping, individualized treatment is beneficial for patients with CNDI caused by different gene mutations. Therefore, the early diagnosis and treatment of CNDI are important. Early interventions can prevent dehydration caused by mental disorders, growth retardation, and other complications. To sum up, gene therapy has become a research hotspot, and advances in genetic research will lead to potential novel treatments for CNDI.

## Data Availability Statement

The datasets presented in this study can be found in online repositories. The names of the repository/repositories and accession number(s) can be found in the article/supplementary material.

## Ethics Statement

The studies involving human participants were reviewed and approved by Lanzhou University Second Hospital. Written informed consent to participate in this study was provided by the participants' legal guardian/next of kin. Written informed consent was obtained from the individual(s), and minor(s)' legal guardian/next of kin, for the publication of any potentially identifiable images or data included in this article.

## Author Contributions

LM collected the data, wrote the manuscript, analyzed the data, and supervised the study. LH participated in the patient's clinical care and collected the data. LL, JL, and TS participated in the patient's clinical care and collected the data. All authors contributed to the article and approved the submitted version.

## Conflict of Interest

The authors declare that the research was conducted in the absence of any commercial or financial relationships that could be construed as a potential conflict of interest.
